# Epidemiology of hypospadias in Europe: a registry-based study

**DOI:** 10.1007/s00345-015-1507-6

**Published:** 2015-02-25

**Authors:** Jorieke E. H. Bergman, Maria Loane, Martine Vrijheid, Anna Pierini, Rien J. M. Nijman, Marie-Claude Addor, Ingeborg Barisic, Judit Béres, Paula Braz, Judith Budd, Virginia Delaney, Miriam Gatt, Babak Khoshnood, Kari Klungsøyr, Carmen Martos, Carmel Mullaney, Vera Nelen, Amanda J. Neville, Mary O’Mahony, Annette Queisser-Luft, Hanitra Randrianaivo, Anke Rissmann, Catherine Rounding, David Tucker, Diana Wellesley, Natalya Zymak-Zakutnia, Marian K. Bakker, Hermien E. K. de Walle

**Affiliations:** 1 Department of Genetics, University of Groningen, University Medical Center Groningen, Hanzeplein 1, P.O. Box 30.001, 9700 RB Groningen, The Netherlands; 2 EUROCAT Central Registry, Centre for Maternal, Fetal, and Infant Research, Institute of Nursing Research, University of Ulster, Northern Ireland, UK; 3 CREAL – Centre for Research in Environmental Epidemiology, Barcelona, Spain; 4 Spanish Consortium for Research on Epidemiology and Public Health (CIBERESP), Barcelona, Spain; 5 CNR Institute of Clinical Physiology, Pisa, Italy; 6 Department of Urology, University of Groningen, University Medical Center Groningen, Groningen, The Netherlands; 7 Division of Medical Genetics, Lausanne, Switzerland; 8 Children’s Hospital Zagreb, Medical School University of Zagreb, Zagreb, Croatia; 9 Department of Hungarian Congenital Abnormality Registry and Surveillance, National Institute for Health Development, Budapest, Hungary; 10 Department of Epidemiology, National Institute of Health, Lisbon, Portugal; 11 Department of Health Sciences, University of Leicester, Leicester, UK; 12 Health Information Unit, Dr Steevens Hospital, Dublin, Ireland; 13 Department of Health Information and Research, Gardamangia, Malta; 14 INSERM U953, Paris, France; 15 Department of Global Public Health and Primary Care, University of Bergen, Bergen, Norway; 16 Centro Superior de Investigación en Salud Publica, Valencia, Spain; 17 South East Ireland Congenital Anomaly Registry, Public Health Department, HSE South (South East), Kilkenny, Ireland; 18 Department of Environment, Province of Antwerp, Antwerp, Belgium; 19 IMER Registry (Emilia Romagna Registry of Birth Defects), Azienda Ospedaliero-Universitaria di Ferrara, Ferrara, Italy; 20 Department of Public Health, Health Service Executive South, Cork and Kerry, Ireland; 21 Universitatskinderklinik Mainz, Mainz, Germany; 22 Unit of Congenital Malformations and Medical Genetics, CHU Reunion, St Pierre, France; 23 Malformation Monitoring Centre Saxony-Anhalt, Medical Faculty, Otto-von-Guericke-University Magdeburg, Magdeburg, Germany; 24 National Perinatal Epidemiology Unit, University of Oxford, Oxford, UK; 25 Congenital Anomaly Register and Information Service Public Health, Singleton Hospital, Wales, UK; 26 University Hospitals Southampton Faculty of Medicine and Wessex Clinical Genetics Service, Princess Anne Hospital, Southampton, UK; 27 OMNI-Net Ukraine and Khmelnytsky Perinatal Center, Khmelnytsky, Ukraine; 28 Medical Birth Registry of Norway, Norwegian Institute of Public Health, Bergen, Norway

**Keywords:** Ascertainment, Congenital anomaly registers, Epidemiology, Hypospadias, Maternal age, Prevalence

## Abstract

**Background:**

Hypospadias is a common congenital malformation. The prevalence of hypospadias has a large geographical variation, and recent studies have reported both increasing and decreasing temporal trends. It is unclear whether hypospadias prevalence is associated with maternal age.

**Aim:**

To analyze the prevalence and trends of total hypospadias, isolated hypospadias, hypospadias with multiple congenital anomalies, hypospadias with a known cause, and hypospadias severity subtypes in Europe over a 10-year period and to investigate whether maternal age is associated with hypospadias.

**Methods:**

We included all children with hypospadias born from 2001 to 2010 who were registered in 23 EUROCAT registries. Information on the total number of births and maternal age distribution for the registry population was also provided. We analyzed the total prevalence of hypospadias and relative risks by maternal age.

**Results:**

From 2001 to 2010, 10,929 hypospadias cases were registered in 5,871,855 births, yielding a total prevalence of 18.61 per 10,000 births. Prevalence varied considerably between different registries, probably due to differences in ascertainment of hypospadias cases. No significant temporal trends were observed with the exceptions of an increasing trend for anterior and posterior hypospadias and a decreasing trend for unspecified hypospadias. After adjusting for registry effects, maternal age was not significantly associated with hypospadias.

**Conclusions:**

Total hypospadias prevalence was stable in 23 EUROCAT registries from 2001 to 2010 and was not significantly influenced by maternal age.

## Introduction

Hypospadias is one of the most common congenital malformations. Hypospadias is often classified into anterior hypospadias: the urethral opening is slightly displaced but still in the glandular or subcoronal region; middle hypospadias: the urethra opens into the ventral surface of the penis; and posterior hypospadias: the urethral opening is located in the penoscrotal junction, scrotum, or perineum [1]. The severity of hypospadias is not only explained by the urethral location, as many other factors (e.g., level of division of the corpus spongiosum, size of the glans, and degree of ventral penile hypoplasia) play a role [2]. Hypospadias may be associated with chordee, cryptorchidism, and other urogenital anomalies. Most patients with hypospadias require surgical correction, but medical, social, and sexual problems may persist after surgery [3].

The etiology of hypospadias is largely unknown. Some hypospadias are monogenetic in origin, but the vast majority of cases seem to be multifactorial with many genetic and environmental factors playing a role [4, 5]. One study found a higher incidence of hypospadias in offspring of vegetarian mothers [6]. However, while several environmental exposures and maternal factors have been studied, only low birth weight, maternal hypertension, pre-eclampsia, and maternal intrauterine diethylstilbestrol exposure have been consistently associated with hypospadias [5]. The association between hypospadias and increased maternal age and exposure to endocrine disrupting chemicals remains controversial [4, 5, 7].

The prevalence of hypospadias shows large geographical variation, ranging from 2.0 to 43.2 cases per 10,000 births [8–10]. It is unclear whether hypospadias prevalence is rising. Early studies reported increasing prevalence [8, 11, 12], while later studies reported increasing [10, 13–15], stable, or decreasing prevalence [16–18]. The varying prevalence and trends might be explained by genetic or environmental risk factors that differ between geographical regions and which increase or decrease locally over time. However, another explanation is methodological differences between studies, i.e., ascertainment of hypospadias cases may vary, and glandular hypospadias and hypospadias with known etiology might be excluded from some studies, explaining the lower prevalence in some regions. Moreover, data on the severity of the hypospadias cases are often unavailable.

In this study, we analyzed the prevalence and trends of total hypospadias, isolated hypospadias, hypospadias with multiple congenital anomalies (MCA), hypospadias with a known cause, and hypospadias severity subtypes in 23 EUROCAT registries from 2001 to 2010. We also investigated whether maternal age was associated with hypospadias prevalence. Our study is the largest of its kind in Europe.

## Patients and methods

### Patient population

EUROCAT registries are population-based, registering data on congenital anomalies in live births, fetal deaths from 20 weeks of gestational age and terminations of pregnancy for fetal anomalies [19]. Cases are actively ascertained from multiple sources, but ascertainment varies considerably due to differences in the data sources that are accessible to individual registries [20]. Of the 31 EUROCAT full member registries, 23 provided data for this study (Table 1; 5,871,855 births covered). The total births covered per year varied from 3040 in Mainz to 100,360 in Hungary.

All participating registries completed a questionnaire concerning their inclusion and ascertainment of hypospadias cases from 2001 to 2010. In 1980, EUROCAT guidelines specified that cases with glandular hypospadias should only be registered if occurring in combination with other major structural malformations [21]. The exclusion of this minor form of hypospadias was to be applied locally. However, Dolk et al. [16] found that some registries were not able to apply the exclusion guideline. In order to ensure consistency and standardization of coding between registries, the exclusion was lifted, and from January 1, 2005, glandular hypospadias cases were included [22].

For this study, data were extracted from the EUROCAT central database in April 2013 for the years 2001–2010. Total hypospadias prevalence rates were calculated as the total number of hypospadias cases divided by the total number of births (male and female) in the population covered by the registry. Hypospadias cases were coded by International Classification of Diseases (ICD) British Pediatric Association; ICD9, codes 7526.01–7526.04 (specified subtypes) and 7526.09 (not otherwise specified, NOS) and ICD10, codes Q54.0–Q54.3 (specified subtypes) and Q54.9 (NOS). Registries were asked to specify all cases with ‘other’ hypospadias (ICD10 code Q54.8, *n* = 68), which led to a better classification in the majority of cases (*n* = 48). The remaining 20 ‘other’ hypospadias cases were excluded from the analyses because of unconfirmed diagnosis. Hypospadias was categorized as isolated cases, MCA cases and cases with a known cause using the EUROCAT MCA algorithm [23]. In isolated cases, no other major structural malformation is present. In MCA cases, hypospadias is seen in combination with at least one unrelated major structural malformation that cannot be explained by an underlying syndrome or sequence. In cases with a known cause, the hypospadias has a chromosomal, genetic, or teratogenic origin. All MCA cases were manually reviewed by the EUROCAT Coding and Classification committee or by a clinical geneticist (JEH Bergman). In two hypospadias cases, the etiology could not be classified after manual review and those cases were excluded.

For the maternal age analyses, we calculated hypospadias prevalence rates within six maternal age groups (Table 2). Hypospadias cases with unknown maternal age (1.5 %) were excluded from the maternal age analyses. Wessex (UK), Hungary, and Isle de la Reunion were excluded as maternal age was missing for >20 % of hypospadias cases or they could not provide maternal age denominators for the population covered by their registry.

### Statistical analysis

Change in the annual prevalence rates over time was assessed by the *χ* ^2^ test for trend and heterogeneity. Results were classified as follows:

- No significant change in prevalence over time: *p* ≥ 0.05 for both linear and nonlinear components;
- Significant increasing or decreasing trend: *p* < 0.05 for trend and *p* ≥ 0.01 for nonlinear change, or *p* < 0.01 for both trend and nonlinear change, and the trend is monotonic;
- Significant heterogeneous in time: *p* < 0.05 for nonlinear change and *p* > 0.05 for trend, or *p* < 0.01 for nonlinear change and *p* ≥ 0.01 for trend.

Multilevel Poisson regression analysis was performed using generalized estimating equations (GEE) to take into account the dependency of cases within registries. In the GEE, the number of cases was corrected for the number of births with year of birth as the predictor.

A Poisson regression model using STATA version 12.1 was used to derive maternal age-specific relative risks (RR) relative to the 25- to 29-year age group baseline. A model for all cases of hypospadias adjusting for registry was fitted to adjust for the possibility that registries with high proportions of mothers in any one age group would bias the RR estimates between age groups.

## Results

### Registration policy

Only four of the 23 participating registries were able to adhere to the original EUROCAT guideline to exclude isolated glandular hypospadias prior to 2005 (Table 1). Fifteen registries had registered all hypospadias subtypes (including glandular hypospadias) from 2001 to 2010, and four registries used different criteria for the registration of hypospadias (Table 1). None of the registries reported a change in case ascertainment specific to hypospadias over the study period. However, case ascertainment in Dublin was affected by data protection issues, and Emilia Romagna increased general ascertainment by adding additional information sources since 2003 and including cases up to 1 year of age (instead of 1 week of age) in 2010. Almost all registries believed that their ascertainment of glandular hypospadias was incomplete, because these cases are not always notified to the registry or they are not diagnosed soon after birth and therefore missed in registries that only have data from the neonatal period. Only Mainz believed that their ascertainment of hypospadias cases was complete, since every newborn is examined by registry pediatricians. Northern Netherlands requires parental consent to register cases, which has been constant at 80 % from 2001 to 2010. Since 2010, the registry also records the diagnosis of cases whose parents did not respond to repeated letters asking for consent. Specification of hypospadias subtype varied considerably between registries, with the percentage of unspecified cases varying between 3.8 and 87.4 %.

**Table 1:** Total number of hypospadias cases, total births, prevalence and 10-year trends in hypospadias, inclusion criteria for hypospadias, and mean percentage unspecified hypospadias per EUROCAT registry in 2001–2010

Registry	Number of hypospadias cases	Total births	Total hypospadias prevalence per 10,000 births	Trend description^c^	*p* value trend	*p* value nonlinear change	Inclusion criteria^d^	Mean percentage unspecified hypospadias^e^
Germany, Mainz^b^	117	31,765	36.83	–	0.371	0.120	2	11.1
Malta	143	39,939	35.80	↓	<0.001	<0.001	3	4.20
UK, Wales	961	333,201	28.84	↓	0.024	0.153	2	34.9
Switzerland, Vaud	192	74,073	25.92	↓	0.021	0.128	3	18.2
Hungary	2426	969,536	25.02	↑	<0.001	<0.001	2	87.4
Norway	1345	593,626	22.66	–	0.123	0.093	2	57.3
Italy, Tuscany	601	292,648	20.54	–	0.148	0.060	2	26.0
Northern Netherlands	375	188,076	19.94	H	0.108	0.008	1	5.6
Ukraine^a^	348	177,149	19.64	↑	<0.001	<0.001	3	7.2
Germany, Saxony-Anhalt	336	174,251	19.28	–	0.613	0.496	2	25.3
Croatia, Zagreb	131	68,283	19.18	↑	<0.001	<0.001	1	3.8
Belgium, Antwerp	345	193,403	17.84	↑	0.047	0.164	2	61.7
Ireland, Cork and Kerry	153	93,015	16.45	↑	0.021	0.017	2	15.0
UK, East Midlands and South Yorkshire	1095	673,844	16.25	↓	<0.001	<0.001	2	86.9
France, Paris^b^	412	267,389	15.41	–	0.718	0.417	2	69.7
France, Isle de la Reunion^a^	199	131,943	15.08	↑	<0.001	<0.001	1	17.1
UK, Wessex	377	279,423	13.49	H	0.485	<0.001	3	41.4
Italy, Emilia Romagna^b^	469	361,324	12.98	↑	<0.001	<0.001	2	33.9
Ireland, Dublin	299	245,851	12.16	↓	<0.001	0.001	2	65.9
Spain, Valencia region^a^	245	217,598	11.26	H	0.222	0.009	2	60.4
UK, Thames Valley	201	204,168	9.84	↑	<0.001	<0.001	2	70.7
South East Ireland	62	71,062	8.72	H	0.107	0.015	1	56.5
South Portugal^b^	97	190,288	5.10	↑	<0.001	<0.001	2	33.0
Total	10,929	5,871,855	18.61	H	0.136	0.013		54.4

^a^Registries with incomplete data: Ukraine only for 2005–2010, Isle de la Reunion only for 2002–2010, Valencia region only for 2007–2010

^b^ Registries that include cases with congenital anomalies diagnosed in the neonatal period and not thereafter (Emilia Romagna: 2001–2009, in 2010 inclusion of cases up to 1 year of age)

^c^ Trend description symbols: ↓ decreasing temporal trend, ↑ increasing temporal trend, H heterogeneous in time, – no significant change in prevalence over time

^d^ Inclusion criteria: 1 = excluded isolated glandular hypospadias prior to 2005 according to original EUROCAT coding guidelines, 2 = registered all types of hypospadias in the entire study period, and 3 = other policy (Malta: 2001–2003 recorded glandular and coronal hypospadias only if surgical correction was planned/performed, 2004–2010 always recorded glandular and coronal hypospadias; Vaud: 2001–2010 recorded glandular hypospadias only if surgical correction was planned/performed; Ukraine: 2005–2007 did not record glandular and coronal hypospadias, 2008–2010 always recorded glandular and coronal hypospadias; Wessex: 2001–2010 recorded glandular and coronal hypospadias only if surgical correction was planned/performed)

^e^ Mean percentage of unspecified subtype of hypospadias 2001–2010

### Prevalence and trends over time

**Fig. 1:**
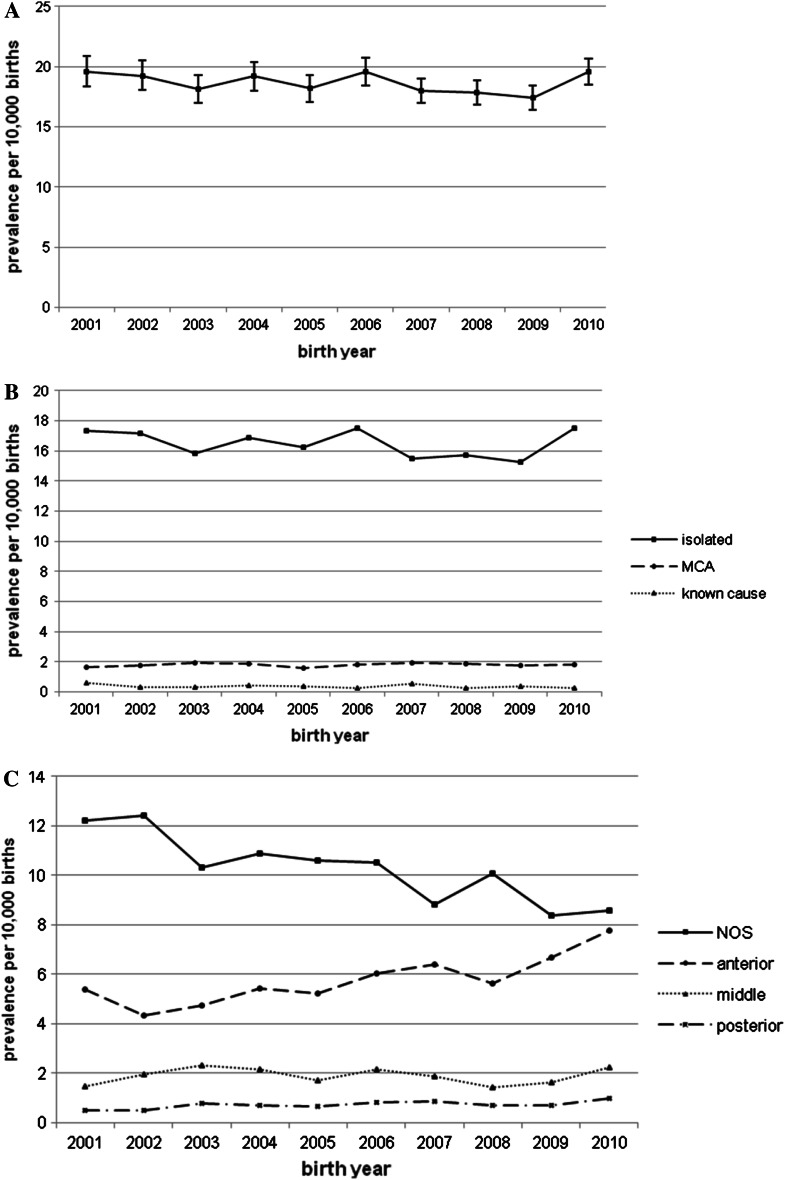
Prevalence of total hypospadias (**a**), isolated hypospadias, hypospadias with multiple congenital anomalies (MCA), hypospadias with known cause (**b**) and hypospadias subtypes (**c**) in 23 EUROCAT registries 2001–2010. **a** All hypospadias: heterogeneous in time (*p* value trend = 0.136, *p* value nonlinear change = 0.013) Multilevel Poisson regression analysis: no significant effect of birth year (*p* value = 0.136) Error bars show 95 % confidence interval. **b** Isolated hypospadias: heterogeneous in time (*p* value trend = 0.137, *p* value nonlinear change = 0.002) Hypospadias with MCA: no significant change in prevalence over time (*p* value trend = 0.576, *p* value nonlinear change = 0.930) Hypospadias with a known cause: heterogeneous in time (*p* value trend = 0.054, *p* value nonlinear change = 0.020). **c** Hypospadias not otherwise specified (NOS): significant decreasing trend (*p* value trend < 0.001, *p* value nonlinear change < 0.001, trend is monotonic) Anterior hypospadias: significant increasing trend (*p* value trend < 0.001, *p* value nonlinear change < 0.001, trend is monotonic) Middle hypospadias: heterogeneous in time (*p* value trend = 0.868, *p* value nonlinear change = 0.001) Posterior hypospadias: significant increasing trend (*p* value trend = 0.005, *p* value nonlinear change = 0.071)

A total of 10,929 hypospadias cases were registered among 5,871,855 births covered by 23 EUROCAT registries from 2001 to 2010, yielding a total prevalence of 18.61 per 10,000 births (Table 1). Prevalence varied considerably per registry, from 5.10 in South Portugal to 36.83 per 10,000 births in Mainz. Total hypospadias prevalence was stable from 2001 to 2010 (*p* value trend = 0.136), but the data were heterogeneous over time (*p* = 0.013) (Fig. 1a). Multilevel Poisson regression analysis confirmed the heterogeneity between registries (*p* < 0.001) but did not show a significant year-of-birth effect (Fig. 1a).

Trend analyses for individual registries showed increasing trends in nine registries, decreasing trends in five registries, heterogeneity over time in four registries, and no significant change in prevalence in five registries (Table 1). Of the four registries that excluded glandular hypospadias following the original EUROCAT coding guideline [21], two had a significant increasing trend, and two were significantly heterogeneous over time (Table 1). In the four EUROCAT registries with the highest hypospadias prevalence, no increasing trend was seen (Table 1).

The majority of hypospadias cases was isolated (*n* = 9667; 88.5 %), 9.6 % were MCA (*n* = 1053), and only 1.9 % of cases had hypospadias with a chromosomal (*n* = 112), genetic (*n* = 86), or teratogenic origin (*n* = 11). There was no significant trend for any of these groups (Fig. 1b).

Hypospadias subtypes were specified in 45.6 % of cases (*n* = 4980); 31.5 % had anterior hypospadias (*n* = 3443); 10.2 % had middle hypospadias (*n* = 1109); and 3.9 % had posterior hypospadias (*n* = 428). For hypospadias NOS, a significant decreasing trend was observed (*p* < 0.001), whereas anterior and posterior hypospadias showed a significant increasing trend (*p* < 0.001 and *p* = 0.005, respectively) (Fig. 1c). Middle hypospadias showed heterogeneous data over time (*p* = 0.001) (Fig. 1c).

### Maternal age and hypospadias prevalence

In this study, teenage mothers had a higher prevalence of total hypospadias compared to mothers aged 25–29 years (unadjusted RR 1.13, 95 % confidence interval (CI) 1.02–1.26, Table 2). This association remained when analyses were repeated excluding chromosomal anomalies. However, after adjustment for registry effects, the increased hypospadias risk in young mothers was no longer significant (adjusted RR 1.12, 95 % CI 1.00–1.24, *p* = 0.051, Table 2).

**Table 2:** Total hypospadias prevalence and relative risk estimates per maternal age category in 20 EUROCAT registries in 2001–2010

Maternal age category (years)	Number of hypospadias cases	Number of births	Total hypospadias prevalence per 10,000 births (95 % CI)	Unadjusted Relative Risk (95 % CI)	Relative Risk adjusted by registry (95 % CI)
<20	385	194,807	19.8 (17.9–21.8)	1.13 (1.02–1.26)	1.12 (1.00–1.24)*
20–24	1333	710,660	18.8 (17.8–19.8)	1.08 (1.00–1.15)	1.06 (0.99–1.13)
25–29	2161	1,239,528	17.4 (16.7–18.2)	1.00 (Reference)	1.00 (Reference)
30–34	2409	1,428,079	16.9 (16.2–17.6)	0.97 (0.91–1.03)	1.00 (0.94–1.06)
35–39	1253	761,353	16.4 (15.6–17.4)	0.94 (0.88–1.01)	0.99 (0.92–1.06)
40+	269	156,496	17.2 (15.3–19.4)	0.99 (0.87–1.12)	1.04 (0.92–1.18)
Total	7810	4,490,923	17.4 (17.0–17.8)		

*CI* confidence interval

Data from Isle de la Reunion, Wessex (UK), and Hungary were excluded. In addition, cases with unknown maternal age (*n* = 117 among 20 registries) were also excluded from maternal age analyses

* *p* value = 0.051

## Discussion

In this large European study, with more than 5.8 million births covered by 23 EUROCAT registries, we found a total hypospadias prevalence of 18.61 per 10,000 births that was stable from 2001 to 2010. Hypospadias prevalence and trends are influenced by the registration policies of the individual registries, as is apparent from the large variations in prevalence and trends per registry (Table 1). This could explain the stable prevalence over time found in this study compared to a previous EUROCAT study reporting an increasing trend over the years 1999–2008 using data from partly overlapping registries [14]. Whether glandular hypospadias is registered or not will have a large effect on total hypospadias prevalence because anterior hypospadias is by far the most common [10, 13]. Ascertainment of hypospadias is incomplete in some registries, and this has a large effect on the prevalence of hypospadias, thus hindering the evaluation of trends. Ascertainment depends on the sources of information that registries can access (e.g., if only surgical data are available, mild cases can be missed), how cases are notified to the registry (active versus passive notification), jurisdiction (data protection issues), differences in diagnostic methods, screening or treatment per country (mild cases might not be diagnosed), and variation in the follow-up period of cases (when follow-up is short, mild hypospadias may not yet have been diagnosed, or a preputial anomaly can be misdiagnosed as hypospadias) [7, 16, 24, 25]. The low hypospadias prevalence in South Portugal (5.10/10,000 births) is largely due to incomplete ascertainment, as ascertainment of all congenital anomalies is low in this registry [26]. The lack of an increasing trend in hypospadias prevalence over all EUROCAT registries combined, as well as in the four EUROCAT registries with the highest prevalence, and therefore probably the best ascertainment, is reassuring.

More reliable prevalence rates and trends can be ascertained by systematically performing standardized examination of complete birth cohorts, as was done in Rotterdam for hypospadias [25] and is routinely done by the Mainz registry. In Rotterdam, a hypospadias prevalence of 38 per 10,000 live births was seen from 1998 to 2000 [25], very similar to the total prevalence of 36.83 per 10,000 births found in Mainz from 2001 to 2010. The main drawbacks of this approach are that the population size is invariably small, only a very limited region is covered, and it is more expensive to conduct.

Although environmental factors are known to contribute to the etiology of hypospadias [4, 5], their role in isolated or MCA hypospadias is largely unknown [18]. In this study, no trends over time were seen in isolated or MCA hypospadias.

Hypospadias cases were also classified according to severity: anterior, middle, or posterior hypospadias. Unfortunately, the severity was known in less than half of hypospadias cases, making interpretation of trends difficult. It is possible that the increasing trends in anterior and posterior subtypes found in this study could be explained by the decreasing trend in hypospadias NOS, as EUROCAT efforts to improve and standardize coding led to better specification of hypospadias by some registries over time.

Increased maternal age has been found to be associated with higher risk of hypospadias in some studies [17, 27, 28], but not in others [15, 29, 30]. In this study, hypospadias prevalence did not differ significantly between the maternal age groups after adjustment for registry, but the highest hypospadias prevalence was seen in teenage mothers and not in older mothers.

This study shows both the advantages and disadvantages of using birth defect registry data to investigate prevalence and trends in hypospadias. While the introduction of new EUROCAT coding guidelines in 2005 was intended to standardize registration, further efforts are needed to implement guidelines locally. We recommend better specification of cases and increased ascertainment where possible. Combining data from birth defects registries with smaller studies that can guarantee complete case ascertainment and classification will provide optimal information about prevalence and trends of hypospadias.

## References

[1] Hennekam RC, Allanson JE, Biesecker LG, Carey JC, Opitz JM, Vilain E (2013). Elements of morphology: standard terminology for the external genitalia. Am J Med Genet A.

[2] Snodgrass W, Macedo A, Hoebeke P, Mouriquand PD (2011). Hypospadias dilemmas: a round table. J Pediatr Urol.

[3] Nuininga JE, de Gier RP, Verschuren R, Feitz WF (2005). Long-term outcome of different types of 1-stage hypospadias repair. J Urol.

[4] Shih EM, Graham JM (2014). Review of genetic and environmental factors leading to hypospadias. Eur J Med Genet.

[5] van der Zanden LF, van Rooij IA, Feitz WF, Franke B, Knoers NV, Roeleveld N (2012). Aetiology of hypospadias: a systematic review of genes and environment. Hum Reprod Update.

[6] North K, Golding J, The ALSPAC study team (2000). A maternal vegetarian diet in pregnancy is associated with hypospadias. BJU Int.

[7] Carmichael SL, Shaw GM, Lammer EJ (2012). Environmental and genetic contributors to hypospadias: a review of the epidemiologic evidence. Birth Defects Res A Clin Mol Teratol.

[8] Paulozzi LJ (1999). International trends in rates of hypospadias and cryptorchidism. Environ Health Perspect.

[9] Toppari J, Kaleva M, Virtanen HE (2001). Trends in the incidence of cryptorchidism and hypospadias, and methodological limitations of registry-based data. Hum Reprod Update.

[10] Nassar N, Bower C, Barker A (2007). Increasing prevalence of hypospadias in Western Australia, 1980–2000. Arch Dis Child.

[11] Czeizel A (1985). Increasing trends in congenital malformations of male external genitalia. Lancet.

[12] Matlai P, Beral V (1985). Trends in congenital malformations of external genitalia. Lancet.

[13] Canon S, Mosley B, Chipollini J, Purifoy JA, Hobbs C (2012). Epidemiological assessment of hypospadias by degree of severity. J Urol.

[14] Loane M, Dolk H, Kelly A (2011). Paper 4: EUROCAT statistical monitoring: identification and investigation of ten year trends of congenital anomalies in Europe. Birth Defects Res A Clin Mol Teratol.

[15] Lund L, Engebjerg MC, Pedersen L, Ehrenstein V, Norgaard M, Sorensen HT (2009). Prevalence of hypospadias in Danish boys: a longitudinal study, 1977–2005. Eur Urol.

[16] Dolk H, Vrijheid M, Scott JE (2004). Toward the effective surveillance of hypospadias. Environ Health Perspect.

[17] Fisch H, Lambert SM, Hensle TW, Hyun G (2009). Hypospadias rates in New York state are not increasing. J Urol.

[18] Martinez-Frias ML, Prieto D, Prieto L, Bermejo E, Rodriguez-Pinilla E, Cuevas L (2004). Secular decreasing trend of the frequency of hypospadias among newborn male infants in Spain. Birth Defects Res A Clin Mol Teratol.

[19] Boyd PA, Haeusler M, Barisic I, Loane M, Garne E, Dolk H (2011). Paper 1: the EUROCAT network–organization and processes. Birth Defects Res A Clin Mol Teratol.

[20] Greenlees R, Neville A, Addor MC (2011). Paper 6: EUROCAT member registries: organization and activities. Birth Defects Res A Clin Mol Teratol.

[21] EUROCAT Guide 1 for the registration of congenital anomalies. (1984) http://www.eurocat-network.eu/content/EUROCAT-Guide-1-1984.pdf. Accessed 2014

[22] EUROCAT Guide 1.3. (2005) Instructions for the registration and surveillance of congenital anomalies. http://www.eurocat-network.eu/content/EUROCAT-Guide-1.3.pdf. Accessed 2014

[23] Garne E, Dolk H, Loane M (2011). Paper 5: surveillance of multiple congenital anomalies: implementation of a computer algorithm in European registers for classification of cases. Birth Defects Res A Clin Mol Teratol.

[24] Nelson P, Nieuwenhuijsen M, Jensen TK (2007). Prevalence of hypospadias in the same geographic region as ascertained by three different registries. Birth Defects Res A Clin Mol Teratol.

[25] Pierik FH, Burdorf A, Nijman JM, de Muinck Keizer-Schrama SM, Juttmann RE, Weber RF (2002). A high hypospadias rate in the Netherlands. Hum Reprod.

[26] Loane M, Dolk H, Garne E, Greenlees R, EUROCAT Working Group (2011). Paper 3: EUROCAT data quality indicators for population-based registries of congenital anomalies. Birth Defects Res A Clin Mol Teratol.

[27] Fisch H, Golden RJ, Libersen GL (2001). Maternal age as a risk factor for hypospadias. J Urol.

[28] Gill SK, Broussard C, Devine O (2012). Association between maternal age and birth defects of unknown etiology: United States, 1997–2007. Birth Defects Res A Clin Mol Teratol.

[29] Loane M, Dolk H, Morris JK, EUROCAT Working Group (2009). Maternal age-specific risk of non-chromosomal anomalies. BJOG.

[30] Materna-Kiryluk A, Wisniewska K, Badura-Stronka M (2009). Parental age as a risk factor for isolated congenital malformations in a Polish population. Paediatr Perinat Epidemiol.

